# Place4Carers: a multi-method participatory study to co-design, piloting, and transferring a novel psycho-social service for engaging family caregivers in remote rural settings

**DOI:** 10.1186/s12913-021-06563-5

**Published:** 2021-06-21

**Authors:** Guendalina Graffigna, Eleonora Gheduzzi, Niccolò Morelli, Serena Barello, Massimo Corbo, Valeria Ginex, Roberta Ferrari, Andrea Lascioli, Carolina Feriti, Cristina Masella

**Affiliations:** 1 Department of Psychology, EngageMinds HUB Research Center, Università Cattolica del Sacro Cuore di Milano, Milan, Italy; 2 Department of Management Engineering, Politecnico di Milano, Milan, Italy; 3 Department of Sociology, Università Cattolica del Sacro Cuore, Milan, Italy; 4 Department of Neurorehabilitation Sciences, Casa Cura Policlinico (CCP), Milan, Italy; 5 Fondazione NEED Institute, Milan, Italy; 6 Azienda Territoriale per i Servizi alla Persona, Vallecamonica, Breno, Italy

**Keywords:** Patient engagement, Caregivers, Co-production, Co-design, Rural setting, Participatory action research, Active healthy ageing, Caregiver burden, Hard to reach populations

## Abstract

**Background:**

Family caregivers are key actors in the ageing society. They are mediators between practitioners and patients and usually provide also essential daily services for the elders. However, till now, few services have been deployed to help caregivers in their care tasks as in improving their mental health which can experience sever burden due to caregiving duties. The purpose of the study is to implement a community-based participatory research project to co-design an innovative organizational model of social services for family caregivers of elderly health consumers living in remote rural areas in Italy.

**Methods:**

This is a community-based participatory research project in the remote area of Vallecamonica involving four main phases. These included a quantitative analysis of caregiver needs, a scoping review on existing services for caregivers, co-design workshops with local stakeholders and caregivers to create a novel service the piloting and a first implementation of the service and the assessment of project transferability to other contexts.

**Results:**

As the hours dedicated to elder care increases, both objective and developmental caregiver’s burden significantly increases. Conversely, higher levels of engagement were associated with lower physical and emotional burden, and caregiver engagement was positively correlated with their perceived self-efficacy in managing disruptive patient behaviours. Based on these preliminary results, four co-design workshops with caregivers were conducted and led to the definition of the SOS caregivers service, built on four pillars structured upon the previous need analysis: a citizens’ management board, training courses, peer-to-peer meetings, and project and service information. We found that co-design is an effective means of creating new services for family caregivers, whose experiential knowledge proved to be a key resource for the project team in delivering and managing services. Less positively, the transferability analysis indicated that local municipalities remain reluctant to acknowledge caregivers’ pivotal role.

**Conclusions:**

A dedicated support service for caregivers can ameliorate caregiving conditions and engagement levels. The service has resulted a successful co-productive initiative for a psycho-social intervention for family caregivers. For the future, we suggest that family caregiver should be considered an active partner in the process of designing novel psycho-social services and not just as recipients to enhance a better aging-in-place process.

## Background

The global ageing population is currently rising dramatically at a rate of 3% each year [1]. There is evidence that environmental factors related to living space and place play an increasingly important role in prolonging human life and, in particular, the quality of life of the elderly [2–4]. ‘Ageing-in-place’—that is, elderly people continuing to live at home for as long as possible—is recognized as a key strategy to improve the quality of life of elderly health consumers and to ensure the sustainability of social and welfare systems. In this scenario, family caregivers play a pivotal role in the daily care of elders, both by assisting with their care and by coordinating interventions and activities with all actors involved in enhancing elders’ health. This requires caregivers to dedicate lot of time to care tasks, which can impact negatively on their own quality of life. For this reason, there is a need for new services to support these caregivers and help caregivers to support older people more effectively by developing evidence-based models. Engaging family caregivers in the care network is potentially a critical asset in implementing ‘ageing-in-place’, especially in remote and rural areas where family caregivers can, if effectively engaged, bridge the gaps caused by the fragmentation of social and welfare systems [5, 6].

Family caregivers arrange and attend medical appointments, participate in both routine and high-stake treatment decisions, coordinate care and services, and help with daily tasks such as dressing, bathing, and managing medicines [7, 8]. Family members have always been the primary source of support and assistance to their relatives in times of illness and when they can no longer function independently [9, 10]. As such, family caregivers constitute an ‘invisible workforce’ within the health care team, but their important role and in particular their support needs often remain unrecognized [10, 11]. Engaging family caregivers in the healthcare process is regarded as a key pillar of improved service effectiveness, sustainability, and patient-centricity [12–16], where engagement is defined as the process of enabling people to become actively and genuinely involved in defining issues of concern to them, in making decisions about factors that affect their lives, in formulating and implementing policies, in planning, developing, and delivering services, and in taking action to achieve change [17].

This paper reports the results of the Place4Cares project [18], a community-based participatory research project to co-designing, piloting and assessing the transferability of an novel organizational model of psycho-social services for family caregivers of elderly health consumers living in the remote rural area of Vallecamonica in Italy. The co-design process was based on an extended review of the literature [19] which clearly evidenced the need for better coordinated intervention dedicated to support family caregivers in hard-to-reach rural area, with a broad scope not only on the improvement of healthcare literacy, but also including psychological support and dedicated spaces to help caregivers’ peers’ networks and collaborations. Moreover, there is a growing agreement in the scientific debate about the opportunity to engaging health consumers and patients in the process of planning and delivering healthcare services. This is even more crucial when actions devoted to promoting psycho-social support are concerned: in this case the involvement of the users of such services is crucial not only to improve their final acceptance of the service but also to guarantee that it is fine-tuned with their expectations and needs. Our project’s aim is to co-produce new services with local service providers and family caregivers to ensure ageing-in-place processes and to strengthen families’ inclusion and engagement in a more effective partnership with the welfare system and local health organizations. An accurate process of caregivers’ inclusion and involvement in all phases of the project was putted in place and critically revised as reported in a previous publication related to the project [20]. In this manuscript, we will report the experiences made in the co-design, first implementation and transferability analysis of the service, to discuss the results achieved and the need for improvements. We will also critically reflect on the lessons learnt for the future.

### The study setting: Vallecamonica

Vallecamonica is a mountainous territory in the northern part of the Lombardy Region. Residential areas in Vallecamonica are geographically dispersed, and the territory’s topography and infrastructural and public transport limitations make service delivery more difficult. On an ageing index (computed as the ratio of people aged 65 or more to those aged 14 or less), the area is characterised by a high proportion of elderly people, with a score of 157.3 compared to the Lombardy Region average of 152.6. This attests to a situation of diffused frailty, with a range of social and healthcare needs. In Vallecamonica, health consumers’ requests for social services are processed by the Azienda Territoriale per i Servizi alla Persona (ATSP); when the agency receives such a request from a health consumer, it evaluates which services to activate and then organizes the delivery through more than 40 providers, which include social cooperatives, foundations, and associations. Data from ATSP’s 2016 Social Balance Sheet show that the number of requests for social services received by municipality social assistants has increased from 2536 in 2012 to 4820 in 2015; about 30% of these were requests for information about home care services, socio-health assistance, and access to nursing home. The number of elderly people receiving home care services increased from 191 in 2012 (29,639 h of assistance delivered) to 235 in 2015 (38,699 h of assistance delivered). While these data show that family caregivers are an important social network node for elderly people living in the area, the increasing demand for health and social services attests to the difficulty (or inability) of family caregivers to support their aged relatives alone.

## Methods

The whole community-based participatory research project in the remote area of Vallecamonica involved four phases (see [18] for a detailed description of the study protocol). In this paper, we shall focus on the results of the Place4Carers project related to the foundation, co-design, first piloting and transferability of the new service (namely: S.O.S. caregivers).

### Phase 1: foundation of the service concept: quantitative analysis of family caregivers’ needs, services usage, and sustained costs

Preliminary, the study involved a quantitative analysis of the caregivers’ need, experiences, and expectations to best orient the co-design phase. This analysis involved two main elements: a quantitative survey and database secondary analysis.

The *quantitative survey* addressed a sample of caregivers whose elders live in Vallecamonica. Eligibility criteria included family caregiver of fragile elders, use of home care services provided by ATSP and four local nursing homes, and residency in Vallecamonica. ATSP representatives were responsible for caregiver recruitment. Because of privacy constraints, ATSP could directly contact only caregivers whose elders were using ATSP home care services. Caregivers whose elders used a home care service organized by one of the four nursing homes were first contacted by their nursing homes. If they were interested in participating in the project, their contact details were shared with ATSP. The survey aimed to measure the psychosocial, economic, and organizational needs of family caregivers and the status of their relatives. To that end, four measures were collected and assessed (see Table 1).

**Table 1:** Interviews with family caregivers: Survey data

Variable of interest	Scales/indicators
Demographics	• Gender • Age • Education • Marital status • Working conditions • Position and sector
Psychosocial	• Caregiving Health Engagement Scale (CHE-s) • Caregiver Burden Scale • Caregiver Self-efficiency Scale • Health Literacy Scale • Caregiver Need Assessment Scale
Organizational	Ad hoc 7-point Likert-type scale assessing caregivers’ satisfaction with existing health and social care services for their elders
Economic	• Out-of-pocket expenditure: medical and non-medical costs, including transportation. • Unpaid time costs

#### Demographic measure

We collected family caregivers’ demographic data, including questions about gender, age, education, marital status, working conditions, position, and sector.

#### Psychosocial measures

To assess the level of caregivers’ needs and engagement, the following widely recognized scales were used.

- *The Caregiving Health Engagement Scale* (CHE-s) investigates the psychological attitudes of caregivers in terms of their participation, competence, and motivation in responding to the care demands of their relatives [13]; measured on a 7-point Likert scale.
- *The Caregiver Burden Scale* measures feelings and well-being when caring for elders [21] on five dimensions: a) objective burden (time-dependent evaluation of stress caused by restrictions on one’s personal life); b) developmental burden (sense of failure regarding one’s hopes and expectations); c) physical burden (physical stress and somatic disorders); d) social burden (caused by role conflicts between job and family); and e) emotional burden (embarrassment or feelings of shame caused by the patient); measured on a 5-point Likert scale.
- *The Caregiver Self-efficiency Scale* assesses caregivers’ sense of self-efficacy in dealing with difficulties related to the care of elders [22], addressing three factors: Obtaining Respite, Responding to Disruptive Patient Behaviours, and Controlling Upsetting Thoughts; measured as a percentage (0–100).
- *The Health Literacy Scale* assesses caregivers’ skills and capacities in understanding and analysing information related to health decisions [23]; measured on a 5-point Likert scale.
- *The Caregiver Need Assessment Scale* explores the needs of family members caring for fragile and vulnerable people [24] in terms of six factors: emotional needs, physical-functional needs, cognitive-behavioural needs, relational needs, social-organizational needs, and spiritual needs (along with a final overall value; measured on a 5-point Likert scale.

#### Organizational measures

To explore caregivers’ satisfaction with existing health and social care services for their elders, we used a selection of ad hoc items on a 7-point Likert-type scale (e.g. ‘*I feel understood by the professional that mainly delivers home treatments’; ‘I am comfortable in sharing my feelings with the professionals that mainly deliver home treatments’; ‘The professionals that mainly deliver home treatments address all my questions and doubts*’).

#### Economic measures

To assess economic strain, we investigated both out-of-pocket expenditure and unpaid caregiver time costs. To measure out-of-pocket expenditure, we used three ad hoc items referring to the average monthly medical and nonmedical costs sustained by the family in caring for elders. 1) ‘*What monthly costs are sustained by caregivers and/or elders for the care of elders in terms of medications, specialized medical examinations, medical aids, special food and beverages, professional caregiver support and others?*’. Among non-medical items, we included transportation costs, calculated as the average price of fuel in Italy [25] multiplied by the total distance travelled by caregivers when accompanying their relatives on clinical visits. 2) ‘*How many journeys do you make every month to accompany your relative on clinical visits?’ ‘What is the average length of these journeys in km?*’).

Transportation costs = avg. price of fuel in Italy x number of journeys accompanying elders x avg. distance per journey].

To assess unpaid time costs, we used one item that estimates the cost of replacing informal caregiver support with professional assistance. 3) ‘*How many hours per month do you spend caring for your relative?*’). More precisely, this replacement cost was calculated as total monthly hours of informal caregiver support multiplied by professional caregivers’ average hourly wage.

Replacement costs = avg. hourly wages of professional caregivers in Italy x number of hours of informal caregiving per month.

A *database analysis* was conducted to integrate the survey results with relevant secondary administrative information about family caregivers’ relatives. Among the elders that used home care services provided by ATSP and four local nursing homes, the analysis included only those whose family caregivers participated in the survey. ATSP and the four nursing homes linked the responses of each family caregiver to their elder’s data, creating elder-caregiver dyads. Elders’ information collected from the ATSP database and the four nursing homes included the following.

#### Status measures

As shown in Table 2, we were interested in elders’ demographic and personal data and information about their clinical condition and service usage characteristics.

**Table 2:** Relatives of interviewed family caregivers: Secondary data sources

Variables of interest	Indicators
Demographic and personal information	• Gender • Age • Education • Yearly income • Number of people living with elder
Clinical condition	• Main pathologies • Civil disability
Service usage characteristics	• Type (and number) of services activated • Type (and number) of activities activated • Number of service hours per month

The analysis of elder-caregiver dyads followed a few pre-defined steps. First, a descriptive analysis was performed to describe the sample of elders and family caregivers [26]. A correlation analysis was then performed using a nonparametric measure (Spearman’s correlation) to identify any significant positive or negative relationships between variables [27]. More precisely, we correlated Caregiver Burden, Caregiver Needs and Caregiver Engagement with the other variables of interest specified in Tables 1 and 2. Given the large number of results, only significant correlations, and those of relevance to this study are highlighted and discussed.

### Phase 2: co-design workshops with caregivers and local stakeholders

Four co-design workshops [28, 29] were conducted to collect ideas and insights for the novel caregiver services. These included a selection of caregivers previously interviewed in the Phase 1 survey in order to 1) explore their caregiving experiences and support needs that existing services in the area fail to meet and 2) co-design a new service to address caregivers’ expectations as articulated in Phase 1. In addition to the co-design process, the workshops invited caregivers to reflect on good practices identified in the Phase 2 scoping review of the literature. The workshops were conducted by two expert moderators (NM, EG), employing a non-directive style to enhance spontaneous participation. Further information can be found in a previously published paper [20] which describes this phase of the research in greater depth.

The workshop transcripts were subjected to qualitative interpretative content analysis [30] to synthesise and map participants’ contributions. Members of the research team performed the analysis as a joint and iterative process. To begin, NM and EG coded the content from the workshops and proposed a preliminary taxonomy. GG, CM, and SB discussed and supervised the first phase of coding and contributed to further synthesis and interpretation. The final synthesis of the workshop results provided a deep description of caregivers’ service needs and expectations, along with a first prototype of the proposed new service. To optimize and finalize the proposal, it was presented for discussion with caregivers in a final workshop. Before proceeding to piloting (Phase 4), the research team presented and discussed the proposed service with the local healthcare organization (ATS della Montagna) and in a dedicated session with the local government committee. The purpose of this workshop was to discuss the feasibility of the new service prototype and to ensure the involvement of local stakeholders in piloting the service.

### Phase 3: piloting and preliminary assessment

Feasible service ideas were implemented through service prototyping [31] in Vallecamonica (Breno, BS) over an 18-month period from April 2019 to November 2020. The pilot was suspended from March to September 2020 because the recent Covid-19 emergency made face-to-face activities impossible, especially for a vulnerable group like elders’ family caregivers. Following a period of adaptation, the service was successfully moved online by October 2020.

Although the target service users were family caregivers and fragile elders using home care services provided by ATSP and Vallecamonica’s four local nursing homes, no exclusion criteria were applied. All meetings were published on the project’s online channels (i.e facebook page, project’s website) and on the ATSP website, enabling everyone interested in the service to join free of charge. ATSP representatives were responsible for caregiver recruitment and implementation of the pilot. As active partners in implementation, family caregivers helped to raise awareness of the project and co-delivered some service activities (e.g. peer-to-peer meetings). The project team organized seven collective meetings (from April to November 2019) in which all members of the project team shared information about the service’s progress and issues arising. At the same time, internal and informal meetings and activities were organised by smaller groups of team members. At the end of the pilot, service outcomes were assessed using both quantitative and qualitative methods; further information about these assessments can be found in a previously published paper [20], which describes the metrics and assessment results in greater depth.

### Phase 4: assessment of transferability to other regions and stakeholder involvement

The final phase of the project assessed the transferability of the Place4Carers project to other remote and rural areas like Vallecamonica and involved the heads of social and welfare service providers in the new territory. Given the particularity of the project setting, we decided to analyse its transferability to the neighbouring territory of Valtellina, which has similar geographical and demographic characteristics, comparable health and social care services and structures for elders, and few services for family caregivers of elderly people (see Table 3).

**Table 3:** Comparison of Vallecamonica with nearby areas

		Local population			Demographic indicators		Geographic indicators	Services structure and		transferability
Comparable areas	City	2018	2008	Variation	Elderly rate	Mortality rate	Population density	Elders living at home	Family caregiver	Comparison
*Vallecamonica*	Breno	4815	5036	−4.39%	216.9	11.6	80.33 ab/kmq	SAD, ADI, RSA Apera	few/no services	Target
*Valtellina*	Sondrio	21,590	22,309	−3.22%	223.2	13.3	1034 ha/kmq	SAD, ADI, RSA Apera	few/no services	similar
	Tirano	9011	9168	−1.71%	212.5	12.1	278.33 ha/kmq	SAD, ADI, RSA Apera	few/no services	similar
	Morbegno	12,405	11,932	3.96%	173.6	11.4	837 ha/kmq	SAD, ADI, RSA Apera	few/no services	similar
*Metropolitan area*	Milano	1,378,689	1,295,705	6%	177.5	10.2	2063 ha/kmq	SAD, ADI, RSA Apera	several services	non similar
*Lake area*	Lecco	337,380	333,460	1%	170.1	9.9	418.8 ha/kmq	SAD, ADI, RSA Apera	some services	non similar

Legend: *SAD* Home-based services, *ADI* Integrated healthcare services, *RSA* nursing home facility

Following a formal presentation of the project, the six heads of social and welfare service providers for vulnerable and elderly people in Valtellina (from Sondrio, Morbegno, Tirano, Dongo, Valchiavenna, and Alta Valtellina) were interviewed using a pre-defined set of questions. These structured interviews included two main sections. The first section explored providers’ perspectives on the transferability of the Place4Carers project by asking them about factors affecting their willingness to implement the project in their area. The second section referred to a Strengths, Weaknesses, Opportunities and Threats (SWOT) analysis performed by the Place4Carers researchers to investigate the extent to which service providers believed that transferring the Place4Carers project to Valtellina would produce the same internal/external achievements and issues as in Vallecamonica. This analysis helped to specify how the outcomes of the Place4Carers project might change in a new area.

All the heads of social and welfare service providers were then invited to join in a participatory workshop to further investigate the transferability of Place4Carers project to their area. Three of the six heads accepted the invitation and discussed how the project should be adapted and modified for new contexts with researchers (*n* = 2), local practitioners (*n* = 4) and politicians (*n* = 1).

The results of the structured interviews and workshop were subjected to qualitative thematic analysis to identify any possible future limiting factors when implementing the Place4Carers project in Valtellina. Triangulation enhanced the reliability of the results.

## Results

### Phase 1: foundation of the service concept: quantitative analysis of family caregivers’ needs, services usage, and sustained costs

Using data from surveys and databases, this phase was devoted to assess the uncovered needs and expectations of family caregivers in their assistential role. The analysis included 51 elder-caregiver dyads (see Table 4).

**Table 4:** Information about elders and their caregivers from ATSP and nursing home databases and survey results

Caregivers				Elders				
		n	%				n	%
***Demographics***					***Demographic and personal***			
Gender	F	40	78%		Gender	F	37	73%
	M	11	22%			M	14	27%
Age	< 55	17	33%		Age	< 70	6	12%
	55–64	17	33%			70–79	11	22%
	> = 65	15	29%			80–89	21	41%
	No answer	2	4%			> = 90	13	25%
Education	Primary School	31	61%		Education	Primary School	37	73%
	High School	19	37%			High School	1	2%
	Higher	1	2%			No response	13	25%
Marital status	Married	43	84%		Yearly Income	Low	20	39%
	Unmarried	1	2%			Medium	9	18%
	Divorced/Separated	4	8%			High	0	0%
	Widower	3	6%			No response	22	43%
Working condition	Employed	15	29%		Number of people living with elder	Alone	21	41%
	Unemployed	16	31%			One person	24	47%
	Retired	19	37%			More than two people	6	12%
	No response	1	2%			No response	1	2%
					Caregivers’s degree of kinship	Husband/wife	6	12%
						Son(s)	35	69%
						Brother/sisters	4	8%
		n	avg.	%		Other	6	12%
***Psychosocial measures***					Service providers	ATSP	31	61%
Caregiving Health Engagement Scale (CHE-s)		51	2.66	67%		Nursing home	20	39%
No answer		3	–		***Clinical condition***			
Caregiver Burden Scale	Time-Dependence Burden	48	16.51	83%	Main pathologies	Cerebrovascular disease	4	8%
	Developmental Burden	48	12.96	65%		Arterial hypertension	8	16%
	Physical Burden	48	10.33	52%		Dementia or Alzheimer	14	27%
	Social Burden	48	5.92	30%		Diabetes type II	8	16%
	Emotional Burden	48	3.22	16%		Heart failure	6	12%
	No response	3	–			Myocardiopathy	7	14%
Caregiver Self-efficiency Scale	Obtaining Respite	47	5.93	59%		Other	4	8%
	Responding to Disruptive Patient Behaviours	47	7.16	72%	Civil invalidity	Yes	47	92%
	Controlling Upsetting Thoughts	47	6.81	68%		No	4	8%
	No response	4	–		Presence of chronic diseases	Yes	30	59%
Health literacy scale		48	1.23	41%		No	21	41%
	No response	3	–		***Service usage characteristics***			
Caregiver Need Assessment Scale		47	28.23	55%	Number of other services activated	Yes	9	18%
No response		4	–			No	40	78%
***Organizational measures***						No response	2	4%
Satisfaction	ATSP	29	5.77	82%	Number of care activities activated	One service	5	10%
	Nursing homes	18	5.71	82%		Two services	22	43%
	No response	4	–			More than two services	24	47%
***Economic measures***					Number of care service hours per month	< 15 h per month	33	65%
	Out-of-pocket expenditure	51	567 €	/month		≥ 15 h per month	17	33%
	Hours of informal caregiving	51	326 h	/month		No response	1	2%
	Unpaid time costs	51	€2296	/month				

#### Overview of elders and family caregivers

The database of assisted elderly in the area of the study included 51 individuals (see Table 4 for demographics). Of these, 53.1% were widowed, and 40% lived alone. Almost 40% earned less than €10,000 per annum and lived alone. In terms of health status, almost 92% were registered as physically impaired, and 60% had at least one chronic disease, including neurological (37.3%), cardiological (27.5%), and other chronic conditions (35.3%). In terms of service demand, 31 (60.8%) were assisted by ATSP, and 20 (39.2%) used nursing home services. Overall, these elders received an average 214.52 ± 140.12 h of assistance each year; those assisted by ATSP received a significantly higher number of hours of assistance (254.17) than those assisted by nursing homes (148.44 h) (*p* = 0.027). The greatest demand was for hygiene and mobility support; almost 90% requested two or more services, and about 55% requested more than three services. About 70% were supported by their sons, and 12% were supported by their husband or wife.

We also analysed the data of 51 family caregivers (see Table 4 for demographics). In relation to caregivers’ wellbeing, almost 60% of the sample reported a moderate or severe level of burden. Regarding their expectations of the social welfare system, caregivers’ main concern was the need for information about local services. Finally, in terms of organizational and economic effort, 40% dedicated more than 70 h per week to caring for their elders, (averaging 75.22 ± 54.14 h per week), and monthly out-of-pocket expenditure for elder support averaged €557. Satisfaction with current home care services and nursing homes was high (82%). Older and unemployed caregivers spent significantly more time caring for their relatives than younger caregivers (*p* = 0.002) or those who were employed (*p* < 0.001).

#### Caregiver burden

Based on our survey data, we analysed the association between demographic factors and caregiver burden (see Table 5). Overall, the level of burden was found to increase with caregiver age. In addition, younger caregivers reported a significantly lower social burden than older counterparts (60–65 years, *p* = 0.016; ≥ 65 years, *p* = 0.016). Social burden also decreased significantly with increased caregiver education (Spearman’s Rho = −.323, *p* = 0.021). Caregivers with primary school education also reported higher levels of physical burden (M = 15.36 ± 2.36) than those with a high school education (*p* = 0.028). Unemployed caregivers reported significantly higher social burden than those who were employed (*p* = 0.005). Caregivers whose elders had activated an ATSP home care service reported higher levels of physical, social, and emotional burden than those assisted by nursing homes (*p* = 0.013, *p* = 0.002, and *p* = 0.032, respectively).

**Table 5:** Correlation between Caregiver Burden and Age and Education^a^

	Physical Burden			Social Burden		Physical Burden	Social Burden	Social Burden	Physical Burden	Social Burden	Emotional Burden
	< 53 vs 54–59 years	< 53 vs 60–65 years	< 53 vs > 65 years	< 53 vs 60–65 years	< 53 vs > 65 years	Primary vs High School	Primary vs High School	Employed vs Unemployed	Nursing home vs ATSP	Nursing home vs ATSP	Nursing home vs ATSP
Mann-Whitney U	30	31	30.5	31	31	30.5	23	130	182	146.5	200.5
Wilcoxon W	108	109	108.5	109	109	240.5	233	250	392	356.5	410
Z	−2.627	−2.38	−2.416	−2.403	−2.402	−2.2	−2.618	−2.822	−2.479	−3.173	−2.55
Sig. Asint.(2 code)	.009^a^	.017^a^	.016^a^	.016^a^	.016^a^	.028^a^	.009^a^	.005^a^	.013^a^	.002^a^	.032^a^

^a^*Only significant and novel correlations are reported here*

As hours dedicated to caring increased, both time-dependence burden (Spearman’s Rho = 0.434, *p* = 0.001) and developmental burden increased significantly (Spearman’s Rho = 0.362, *p* = 0.009). Caregiver burden did not differ significantly by gender, family role, or elder’s age, pathology, or living arrangement (see Table 6).

**Table 6:** Correlation between Caregiver Burden and monthly hours of informal caregiving^a^

		Time-Dependence Burden	Developmental Burden	Physical Burden	Social Burden	Emotional Burden
N. hours of informal caregiving	Correlation Coeff.	.434	.362	0.197	0.146	0.223
	Sig. (2-code)	.001^a^	.009^a^	0.166	0.307	0.115
	N	51	51	51	51	51

^a^*Only significant and novel correlations are reported here*

#### Caregiver needs

Our results show that the spiritual needs of caregivers increase significantly with increasing age (Spearman’s Rho = .455, *p* = 0.002) among those aged 54–59 (*p* = 0.005), 60–65 (*p* = 0.008), and ≥ 65 (*p* = 0.006) (see Table 7). Caregiver schooling also influences the extent of expressed needs, with an observed decrease in total (Spearman’s Rho = −.325, *p* = .026), cognitive (Spearman’s Rho = −.303, *p* = .039), relational (Spearman’s Rho = −.352, *p* = .015) and spiritual needs (Spearman’s Rho = −.291, *p* = .047) as years of schooling increase. Caregivers of elders who used ATSP services had greater needs than those caring for elders assisted by nursing homes, both overall (*p* = 0.014) and on the subscales of emotional (*p* = 0.026), cognitive-behavioural (*p* = 0.012), and relational needs (*p* = 0.016) (see Table 7 for further information on correlations).

**Table 7:** Correlations between Caregiver Needs and Caregiver Age and the living condition and type of services of their relatives^a^

	Spiritual needs			Relational needs	Spiritual needs	Total Caregivers needs	Total Caregiver needs		Total Caregiver needs
	< 53 vs 54–59 years	< 53 vs 60–65 years	< 53 vs > 65 years	Primary vs Secondary Schools	Primary vs Secondary Schools	Employed vs Unemployed	Live alone vs with partner	Live alone vs with son /daughter	ATSP vs rest house services
Mann-Whitney U	27.5	33	27.5	124.5	27.5	118	19	41.5	152.5
Wilcoxon W	93.5	99	93.5	334.5	237.5	196	74	86.5	342.5
Z	−2.806	−2.641	−2.775	−2.238	−2.278	−2.153	−3.492	− 2.166	−2.463
Sig. Asint.(2 code)	0.005^a^	0.008^a^	0.006^a^	0.025^a^	0.023^a^	0.031^a^	0^a^	0.03^a^	0.014^a^

^a^*Only significant and novel correlations are reported here*

In general, the greater the expressed needs, the lower is perceived self-efficacy (*p* = 0.005). Furthermore, the lower the self-efficacy in managing patients’ destructive behaviours, the greater are total (Spearman’s Rho = −.356, *p* = 0.016), emotional (Spearman’s Rho = −.603, *p* < .001), social (Spearman’s Rho = −.354, *p* = 0.017) and relational needs (Spearman’s Rho = −.311, *p* = 0.038). Additionally, difficulty in reading and interpreting medical information as measured by the Health Literacy Scale increases as total expressed needs increase (*p* = 0.027). Increased cognitive-behavioural and relational needs are associated with greater caregiver difficulty (Spearman’s Rho = .354, *p* = 0.018, and Spearman’s Rho = .353, *p* = 0.019, respectively). As physical burden increases, total (*p* = 0.002), physical-functional (*p* = 0.01), cognitive-behavioural (*p* = 0.045), relational (*p* = 0.032), social (*p* < 0.001) and spiritual (*p* = 0.027) needs increase (see Table 8 for more details on correlations).

**Table 8:** Correlation between Caregiver Total Needs and Self-efficacy, Burden and Health Literacy^a^

		Responding to Disruptive Patient Behaviours	Physical burden	Social burden	Health literacy
Total Caregiver needs	Correlation Coeff.	−.356	.445	.528	.332
	Sig. (2-code)	0.016^a^	0.002^a^	0^a^	0.027^a^
	N	45	47	47	44

^a^*Only significant and novel correlations are reported here*

#### Caregiver engagement

No significant differences were found in engagement as assessed by the Caregiving Health Engagement Scale by age, gender, education (both caregiver and elder), caregiver employment status, or hours of assistance required. Higher levels of engagement were associated with less physical (Spearman’s Rho = −.333, *p* = 0.019) and emotional burden (Spearman’s Rho = −.469, *p* = 0.001) (see Table 9). Caregiver engagement correlated positively with caregivers perceived self-efficacy in managing disruptive patient behaviours (Self-Efficacy Scale, Spearman’s Rho = .568, *p <* 0.001) and negatively with needs as assessed by the Caregiver Need Assessment. Higher engagement was associated with lower total (Spearman’s Rho = −.339, *p* = 0.021), emotional (Spearman’s Rho = −.398, *p* = 0.006) and spiritual needs (Spearman’s Rho = −.305, *p* = 0.04).

**Table 9:** Correlation between Caregiver Health Engagement and Caregiver Burden, Self-efficacy and Caregiver Needs^a^

		Physical Burden	Emotional Burden	Responding to Disruptive Patient Behaviours	Emotional needs	Spiritual needs	Total Caregiver Needs
Total Caregiver Health Engagement	Correlation Coeff.	−.333	−.469	.568	−.398	−.305	−.339
	Sig. (2-code)	0.019^a^	0.001	0^a^	0.006^a^	0.04^a^	0.021^a^
	N	49	49	48	46	46	46

^a^*Only significant and novel correlations are reported here*

### Phase 2: co-design workshops with caregivers and local stakeholders

In total, 26 of the caregivers who participated in Phase 1 and 7 stakeholders that participated in the following part of the project (see Table 10) have been involved in co-design workshops to generate insight for the novel service devoted to support family caregiver engagement. The great majority were females in their sixties, mainly retired from work, with a low level of education.

**Table 10:** Sample characteristics

Variable	%
Gender	
Female	72
Male	28
Age	
40–49	16
50–59	20
60–69	52
≥ 70	12
Marital status	
Unmarried	8
Cohabiting	4
Married	80
Divorced	8
Level of school attainment	
Low	56
Middle	40
High	4
Employment status	
Unemployed	20
Retired	48
Housewife	12
Full time job	12
Part-time job	8

(*n* = 25)

#### Need for information

According to the co-design workshops’ results, caregivers report great difficulties in accessing information about services, benefits, initiatives, and bureaucratic procedures. They attribute this lack of information to a lack of clear sources of reference for medical information in this area (e.g. lack of general practitioners) and to the extreme effort (in terms of energy and time) needed to collect and organize the relevant information. Interviewees noted the need for a single user friendly and constantly updated source of information about medical, social, legal and practical aspects of caring for older people. They also highlighted the need for a clear map that would enable them to locate dispersed services in the territory. *‘The problem here in the valley is access to information; we do not know how to find information about procedures or facilities’ (Caregiver 5, female). ‘It would be useful to have a job description detailing what the doctor does and where he does it, and also a person dedicated to helping caregivers who may not be comfortable with online services’ (Caregiver 1, female)*. Reference was also made to the need for a website and telephone line with trained volunteers to address questions and doubts: *‘Especially when looking after someone with Alzheimer’s, we need a help desk with a dedicated phone number and someone that can help us emotionally as well as with information’ (Caregiver 8, female)*. This information system should be shared with general practitioners and social workers, as *‘they are extremely helpful and careful, but they too have great difficulties in finding all the necessary information’ (Caregiver 12, male).*

#### Best practices for sharing needs

A second need identified by caregivers was to learn best practices for supporting their relatives. Even those who gained such expertise during an extended period of caregiving showed many difficulties in this regard. The general feeling was that, rather than proceeding by ‘trial and error’, they would welcome advice, counselling, and guidelines to improve their caring capabilities and to save time. Caregivers reported several examples of situations where they felt unprepared. *‘My mother has Alzheimer’s, and I don’t know what to do. For example, sometimes she asks me where her mum is, who died years ago. What am I to do? Should I indulge her? What are the risks of doing that?’* (Caregiver 4, Female). *‘The problem is that we are not prepared to face the illness. We find ourselves in a difficult position because we do not know how to act when a person has Alzheimer’s; we recognize the illness, but what is and is not appropriate? When you encounter the situation directly, that’s another story’* (Caregiver 16, male).

For the interviewed caregivers, the experience of feeling inadequate and lacking in solid expertise is a major factor in the potential emotional burden, and they reported a need *‘to have a proper training, not only to deal with the illness but also to learn about practical issues and how to help our relatives in their everyday life’* (Caregiver 19, male).

#### Emotional needs

The emotional burden of caregiving was referred to repeatedly during the workshops, along with the need for spaces and occasions where these feelings can be expressed, with some form of empathetic listening and support. *‘To be honest, I feel like I’m in prison since I began to look after my mother. We are at risk; I realise that I can become angry and very nervous’* (Caregiver 20, male). *‘I think we should organize as a group to share our experiences; WhatsApp is ok, but a proper physical encounter is more important’* (Caregiver 9, female). All participants agreed about the need to express and share their emotional burden with peers. *‘A group where I can share how I feel, not feel abandoned, and share our experiences and moments together’* (Caregiver 2, female). Finally, thanks to the previous positive experiences of some caregivers, the importance of psychological counselling for emotional support also emerged. *‘I found great comfort in a previous group at the hospital, where a psychologist helped us to find emotional support and relief. I think that is also important for reducing our burden’* (Caregiver 2, female)*.*

### Phase 3: piloting and preliminary assessment

#### Service co-design

The researchers and ATSP personnel discussed the service ideas proposed by family caregivers in the co-design workshops in phase 2 (Tables 11 and 12) putting them in reference to what emerged from the quantitative need analysis conducted in phase 1 and attempted to put them into practice.

**Table 11:** Service ideas proposed by family caregivers in the co-design workshops

Service ideas	Economic feasibility	Organizational feasibility	Major constraints	Implementation
Help desk	low	low	Insufficient project budget and resources	not implemented
Dedicated website informing caregivers and relatives	medium	high		**implemented**
Information brochure	high	high		**implemented**
Green line dedicated to support caregivers	low	low	Insufficient project budget and resources	not implemented
Information about available services	high	high		**implemented**
Online group for sharing concerns	high	medium		**implemented**
Additional support hours for caring activities	low	low	Insufficient project budget and resources	not implemented
Additional professional support hours/days for relatives	low	low	Insufficient project budget and resources	not implemented
Training and general practitioner involvement	high	low	Bureaucratic issues blocked course delivery for general practitioners	not implemented
Training courses	high	medium		**implemented**
Caregivers time bank	high	low	High caregiver burden and caring time demands prevented implementation. However, a self-help group time bank was incentivized.	***partially implemented***
Psychological support	low	medium	As one-to-one psychological support was too costly, the psychologist got involved in the self-help group.	***partially implemented***
Self-help groups	high	high		implemented
TV commercial	low	high	The project budget was too limited to fund a TV commercial. However, the project team delivered three interviews on local media	***partially implemented***
Busto transport elders to local hospitals and ambulatories	low	low	Insufficient project budget and resources	not implemented

**Table 12:** Structure of SOS Caregivers service

	SOS Caregivers		
**Organizational structure**	Health consumers’ management board		
**Service activities**	Training programme	Peer-to-peer meetings	Project and service information

Activities that were considered feasible in economic and organizational terms were included in the new service pilot, which was called *SOS Caregivers*. The service was built on four pillars: a health consumers’ management board, training courses, peer-to-peer meetings, and project and service information. While the last three of these were based on caregivers’ explicit suggestions, the health consumers’ management board was proposed based on caregivers’ willingness to play an active part in service design, delivery, and assessment. During the design phase, participants enjoyed collaborating and requested further opportunities to be involved in developing the project and ensuring its effectiveness. ‘*We have left our email and phone contacts here, and [we will wait for your email or phone call in the coming months]*’(Caregiver 6, woman). ‘*Are you [service providers and researchers] going to contact us in the months ahead?*’(Caregiver 9, woman). ‘*But do we have to implement all these ideas by ourselves in the coming months?*’ (Caregiver 4, man). ‘*[..] Or must we wait for you?*’ (Caregiver3, woman).’

The health consumers’ management board included ATSP representatives, researchers, and family caregivers (representing the great majority of board members). The board was open to any family caregivers who were interested in joining. The board had two purposes: to support and advise ATSP in implementing service activities, and to give family caregivers a voice and responsibility. The members met every 4 months to discuss service issues and possible improvements. All members had an equal say in final service decisions; in fact, the number of family caregivers at board meetings was always (at least) double the number of researchers and ATSP representatives. The direct contributions of family caregivers to the management and evaluation of service activities helped ATSP personnel and the researchers to understand caregivers’ needs and preferences [32]. The involvement of family caregivers also helped to build strong relationships [33] with ATSP and a shared sense of community [34].

*The training programme* provided a set of practical courses for family caregivers to help them to care for their relatives. Enhancing caregivers’ skills and capabilities has a positive impact on the quality of elder care and the well-being of elders and caregivers alike. Courses were organized monthly and were delivered by professionals including psychologists, social workers, educators, speech therapists, and physiotherapists. Course content reflected the needs and difficulties highlighted by family caregivers during the co-design workshops. Five courses were face-to-face (helping relatives to swallow, supporting relatives during routine activities, dealing with stressful situations, preventing relatives’ falls, and managing relatives’ medications), and two were delivered online (sanitary best practices in elder care, dealing with elders during the Covid-19 pandemic). At the end of each course, the professionals’ material was shared with participants.

*Peer-to-peer meetings* were attended by family caregivers and coordinated by one psychologist. At the beginning of each group meeting, the psychologist suggested a theme related to the caregivers’ daily life and encouraged participants to express their opinions and experiences in this regard. The aim was to create a self-help network of caregivers, enhancing their sense of well-being and belonging to a community by sharing ideas. During these group meetings, the psychologist sought to promote equal and fair participation, and caregivers were asked to support the psychologist in co-delivering the service activity. While the psychologist acted as moderator, caregivers were responsible for developing the group discussion. The self-help meetings during the service pilot (five face-to-face and two online) encouraged serious reflection as well as more general discussion about the role of caregivers, managing one’s private life, cultural and culinary habits in Vallecamonica, memories related to family life, and the difficulties of living in remote and rural areas.

*Project and service information* provided caregivers with the information they needed in three ways (see Table 13). First, with the support of the four local nursing homes, the project team produced a report summarizing the bureaucratic procedures, admission constraints, costs, and activities associated with services in Vallecamonica for elderly people living at home. Second, caregivers were informed about the new service activities during the pilot. Finally, one psychologist launched a WhatsApp group to facilitate direct communication with caregivers who had expressed their interest. These three formal and informal sources were complementary; while the report provided information about available activities for elderly people, the service activities kept people informed about opportunities for supporting caregivers, and the WhatsApp group collected caregivers’ other informal requests for support.

**Table 13:** Service activities and achievements

	SOS Caregivers		
**Organizational structure**	Health consumers’ management board ***Number of activities:*** 3 meetings ***Average number of participants per meeting:*** 7 caregivers, 2 ATSP representatives, 2 researchers		
**Service activities**	Training programme Total participants: 39	Peer-to-peer meetings Total participants: 29	Project and service information
**Number of activities between** **April 2019–February 2020**:	5 training sessions (+ 1 postponed): 1. *Helping relatives with swallowing* 2. *Supporting relatives in routine activities* 3. *Dealing with stressful situations* 4. *Preventing falls and managing medications* 5. *Implementing sanitary best practices*	5 group meetings (+ 1 postponed): 1. *The role of caregivers* 2. *Managing caregivers’ private life* 3. *Cultural and culinary habits of Vallecamonica* 4. *Memories related to family life* 5. *Difficulties of living in remote and rural areas*	1. Project website and Facebook page 2. Brochures about the service 3. ATSP website – news section 4. WhatsApp group coordinated by ATSP 5. Report summarizing service information for relatives in Vallecamonica
**Number of online activities between** **October–November 2020**	2 online training sessions 1. *Helping relatives with swallowing* 2. *Dealing with relatives during the Covid-19 pandemic*	2 online group meetings: 1. The effects of Covid-19 in daily life 2. How to deal with more complex and stressful situations	Two online recordings of training sessions;
**Average number of participants per session/meeting**	7 caregivers 1 formal teacher 1 ATSP representative	6 caregivers 1 psychologist	83 Facebook page followers > 130 visitors to the project website
**Average satisfaction**	98%	86%	–

To reach as many recipients as possible, the report and service activities were disseminated through a new project website (https://www.place4carers.it/), a project Facebook page (https://www.facebook.com/place4carers), brochures, and alerts in the news section of the ASTP website and newsletter.

#### Service activities

Overall, Place4Careres – as a whole project- reached in the different phases of the research and intervention more than 150 family caregivers. Regarding the service, it involved a total of 69 caregivers (see Table 13 for more details about the contents and the numbers of participants of each activity). We think that at least two external constraints limited attendance. First, caregivers’ initial mistrust meant that few participants were willing to participate in the first training session and group meeting, forcing the project team to postpone those activities. Second, caregivers’ low digital literacy contributed to the low number of participants in the first online training sessions and group meetings (*n* = 0 online, *n* = 3 offline). Despite these setbacks, overall satisfaction with service activities was very high, confirming their positive effect on caregivers’ well-being.

For more information about the qualitative and quantitative analyses of the service pilot, see [20].

#### Supporting activities

**Fig. 1:**
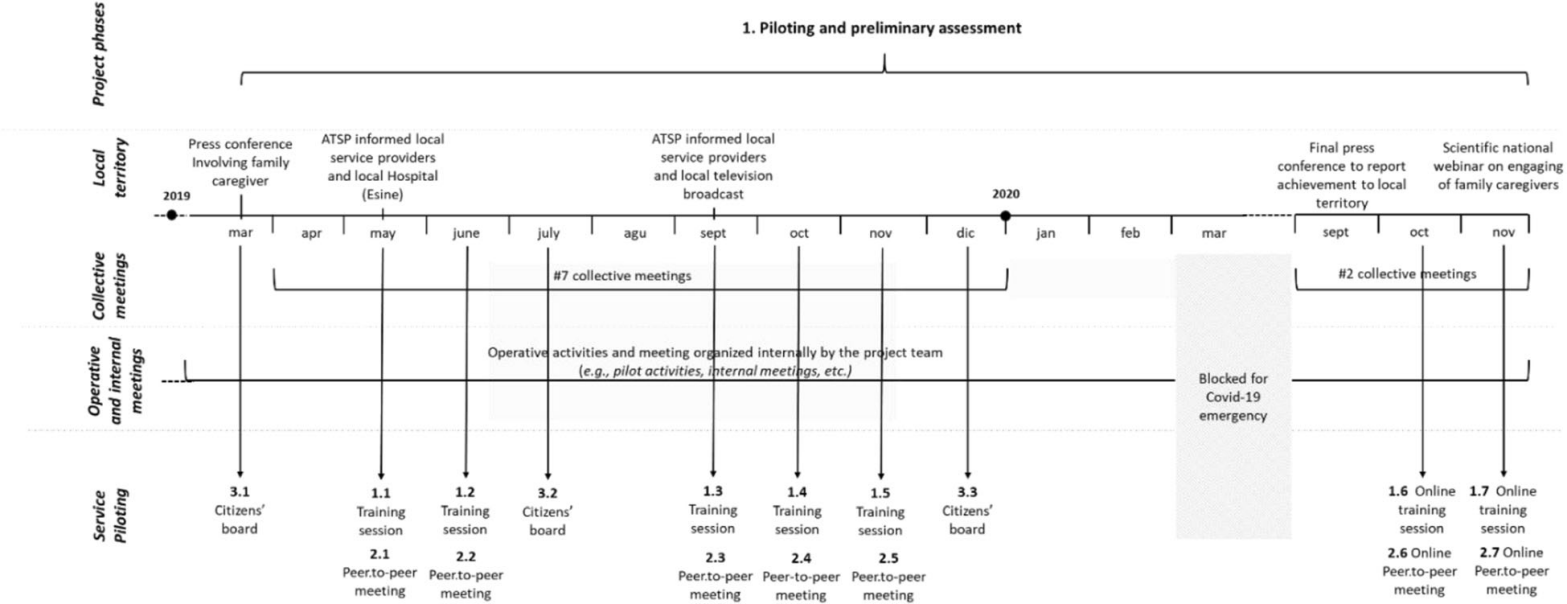
Internal and external activities during the pilot and preliminary assessment phase

The service was implemented along with supporting activities that ensured its success (Fig. 1). At the launch and again at the end of the pilot, an account of the service was presented to the local health agency (ATS della Montagna) and a government committee, and a brief overview was disseminated through local newspapers and broadcast media. During the pilot, ATSP and the project team raised awareness of the service through presentations to local service providers, including the local hospital, nursing homes, cooperatives, and social workers, and an official communication was released on local broadcast media. Additionally, the project team organized seven collective meetings and several internal operational meetings to manage and oversee project progress.

### Phase 4: assessment of transferability to other regions and stakeholder involvement

The research team reflected on internal/external achievements and issues arising throughout the service pilot in Vallecamonica in order to assess the potential transferability of the service to other contexts. More precisely, the following issues were addressed.

- Direct achievements: factors that impacted directly on family caregivers or service providers (e.g. increased levels of trust between caregivers and service providers)
- Indirect achievements: actual or potential indirect impacts on one or more actors in the service ecosystem (e.g. increased motivation of service providers following direct collaboration in co-producing a new service)
- Internal issues: service requirements that negatively affected (or threatened to affect) family caregivers or service providers (e.g. time and effort invested in managing and delivering the service)
- External issues: external factors that affected (or threatened to affect) one or more actors in the service ecosystem (e.g. participation difficulties when caregivers were unable to move their relatives and/or leave them alone at home).

The research team then sought to determine whether the identified achievements and issues might transfer to the new service setting (Valtellina). To that end, we interviewed the heads of three social and welfare service providers who had made themselves available. All three felt the Place4carers project was useful and interesting for their district because it would improve caregivers’ wellbeing and the effectiveness of relative’s support. They also welcomed the active involvement of family caregivers in the service life cycle as a means of enhancing trustful relationships between caregivers and service providers, establishing a peer-to-peer community, and disseminating existing health and social care services. All three felt that equal collaboration would have no negative effects on caregivers or service providers—that is, caregivers would not feel useless, and professionals would not lose their authority or control. All three believed that caregivers would be interested in participating in the SOS Caregivers service, and that local stakeholders in Valtellina (e.g., nursing homes) would support this new service. However, two of the three interviewees expressed concern that project implementation might demand undue effort and resources, and only two felt that the project would increase the motivation of professionals and caregivers in relation to caring activities.

To integrate and deepen these preliminary results, researchers organized workshops involving the three heads of social and welfare service providers and their key actors, including local social workers, cooperatives, and political parties working with the social care services. During the discussion, at least two limiting factors emerged that would require service providers to modify the Place4Carers project prior to implementation in Valtellina. First, despite its relevance, participants believed that it was premature to invest in services for family caregivers, and that new services should target both caregivers and relatives. Most elderly people living in Valtellina do not receive appropriate health and social care support because the limited economic and human resources dedicated to caring activities cannot meet service demand in time. For these reasons, workshop participants argued that family caregivers should not be the only target for new services; in their view, relatives’ needs are the root cause of caregivers’ discomfort, and that strain can therefore be relieved by supporting services for relatives.

Secondly, participants reflected on the difficulty of integrating and coordinating service activities in Valtellina. In the area managed by the three social and welfare service providers, the list of stakeholders supporting elderly people is long (21 nursing homes, five cooperatives, and three volunteering organizations), and the service network is complex to manage. For that reason, participants believed that Valtellina’s existing home care service network should be reinforced by increasing cohesion among stakeholders before launching Place4Carers project, which requires different stakeholders to collaborate.

## Discussion

Scholars have described family caregivers as the invisible backbone of the social and welfare systems [18]. As well as providing daily assistance, family caregivers play a pivotal role in linking and integrating the various actors and services that support elderly health consumers. This is particularly evident in remote and rural areas, where family caregivers can, if effectively engaged, bridge the gaps caused by the fragmentation of the social and welfare systems. According to our data, 40% of caregivers dedicate more than 70 h each week to their relatives; average monthly out-of-pocket expenditure on supporting relatives is €556; and satisfaction with existing services is high (home care services: 90%; nursing homes: 80%). These findings align with previous evidence that family caregivers play a key role in Western integrated care [34], and that caregiving overload has negative psycho-social consequences [35]. Specifically, as the hours dedicated to elder care increase, both objective and developmental burden increase significantly. Caregiver burden did not differ significantly by gender, family role, user age, pathology, or living arrangements. Our analysis of the association between caregiver burden and needs revealed that higher levels of social burden are associated with significantly greater needs, again aligning with previous evidence from other cultural and healthcare contexts [36]. Conversely, higher levels of engagement were associated with lower physical and emotional burden, and caregiver engagement was positively correlated with their perceived self-efficacy in managing disruptive patient behaviours. This tends to confirm that family caregiver engagement is especially crucial for medically frail patients (e.g. children, elderly people affected by mental disorders or neurodegenerative disease) [37] and those living in rural or remote communities, where healthcare services are often fragmented and geographically dispersed. Failure to support active engagement among family caregivers may therefore be regarded as a missed opportunity for ensuring the sustainability of healthcare services and the effectiveness of clinical relationships [38] and all medical interactions. To date, however, little is known about the unique needs of elderly health consumers’ family caregivers or their expectations regarding health and care services, and little attention has been devoted to their perspectives and communication needs in the healthcare environment, especially in rural and remote areas.

The present findings also confirm that external support is entirely insufficient to meet the needs of service users, and professional caregivers are often required to fill gaps in service users’ care needs. This aligns with evidence from other studies regarding the crucial role of family members in caring for elderly patients [39, 40]. The quantitative analysis further confirmed that female individual are the pivotal family caregivers, providing both pragmatic and emotional support for their relatively relatives. Again, this finding aligns with previous evidence that the caregiving burden is typically borne by women [38, 40]. Female caregivers were also the main participants in the service co-design and early implementation phases of this project, confirming existing evidence that informal caregiving falls mainly to wives or daughters, who devote much of their life to caring tasks [38]. In a remote rural area like Vallecamonica, patterns of caregiving are also affected by the fact that young people tend to gravitate to more populated areas. The more highly educated and those looking for better jobs are motivated to escape while people with lower levels of education tend to remain in the valley, and care duties therefore fall to them. As the scoping review indicated, there are few services to support family caregivers in rural and remote areas despite calls for more research in this regard [41]. Lack of resources, high service demand, and low service accessibility mean that local service providers invest fewer resources in services for relatives, and this is the root cause of caregivers’ strain. What also emerged from the study was the clear need for peer-to-peer groups to allow caregivers to share their experiences in the pursuit of empathy and relief. As in other social contexts, it also appears that while technology can provide significant logistical support, people still need human encounters that cannot be provided digitally [19].

The present research demonstrates that family caregivers can and should play an active role in the design, delivery, and assessment of new elder services. Recent studies have highlighted several benefits of co-production as ‘an umbrella concept that captures a wide variety of activities that can occur in any phase of the public service cycle and in which state actors and lay actors work together to produce benefits’ [42]. First, the study confirms that co-production increases the quality of services and outcomes because final solutions are designed and created on the basis of user needs and requests [43]. Secondly, co-production enhances user satisfaction and use of co-productive solutions [44, 45]. Third, close collaboration increases trust among users, service providers, and service organizations by fostering strong and trustful relationships [46]. Finally, close collaboration with service users taps into different points of view, increasing the level of service innovation [47]. In line with previous scientific evidence, we found that co-production is an effective means of creating new services for family caregivers, whose experiential knowledge proved to be a key resource for the project team in delivering and managing services [48]. In the co-design phase, caregivers drew on their personal daily experiences to identify the most useful ideas. In the co-delivery and co-assessment phases, they provided timely feedback on service effectiveness by reporting their personal experiences of service activities. The practical insights and suggestions of family caregivers enabled service providers to shape effective satisfactory, and successful services that made caring activities more effective. Recognizing their crucial role, family caregivers sought to be treated as partners in elder care. To that end, they sought to increase their technical knowledge and competences by requesting training courses and informational materials that would help them to become more effective and knowledgeable in managing and delivering care. In short, investing in caregivers’ knowledge is likely to increase the effectiveness of elder care and support [49].

Less positively, the transferability analysis indicated that local municipalities remain reluctant to acknowledge caregivers’ pivotal role. Given the increasing number of elders and difficulties in caring for them, policy makers prefer to address elders’ needs directly. However, as the Covid-19 emergency confirms, this model of care delivery may fail because resources are limited and demand is rising [50]. In these circumstances, valuing caregivers’ support may offer a new and sustainable model of care that reduces the medium- and long-term strain on health and social care facilities [51]. Future studies should seek to confirm the relevance of caregiver engagement in all phases of the service life cycle.

## Conclusions

The paper describes the foundation, co-design, first piloting and transferability analysis of a novel psycho-social support service dedicate to family caregivers of elderly people in the remote rural area of Vallecamonica. The process itself can be considered a good practice for innovating the healthcare and welfare support dedicated to aging in place initiatives. Indeed, moving for the deep analysis of the population characteristics, of their caregiving burden, service usage levels and expectations of support was then possible to directly engage family caregivers in the co-design of a new social service able to truly answer their needs and expectations, in a full sensitiveness to the cultural and anthropological specificities of their local community. The SOS Caregivers service described here is based on the idea that a dedicated support program can enhance caregivers’ ability to care more effectively for elderly health consumers and to become valuable partners in the social and welfare systems. In other words, family caregivers should be seen as active partners in elder care rather than as mere recipients, and policy makers and researchers should involve family caregivers in decision-making about relatives’ care and services, acknowledging the value of their support.

### Limitations

Future research should investigate the contextual, economic, demographic, and geographic factors that warrant investment in elder care rather than caregiver support. As the present analysis is context-specific, the results are less generalizable, and further research should investigate the effects of caregiver engagement in other settings, such as urban areas and developing countries. Finally, privacy issues made it impossible to access clinical data, which prevented comparison of patient-caregiver dyads.

## Data Availability

The datasets used and/or analysed during the current study available from the corresponding author on reasonable request.
